# *BIM*基因多态性与复治晚期非小细胞肺癌EGFR-TKI治疗疗效的关系

**DOI:** 10.3779/j.issn.1009-3419.2013.12.03

**Published:** 2013-12-20

**Authors:** 蕾 郑, 宝钗 林, 正波 宋, 发君 谢, 卫 洪, 建国 冯, 岚 邵, 沂平 张

**Affiliations:** 1 310053 杭州，浙江中医药大学第二临床医学院 The Second Clinical Medical College, Zhejiang Chinese Medical University, Hangzhou 310053, China; 2 310022 杭州，浙江省肿瘤医院化疗中心 Department of Chemotherapy, Zhejiang Cancer Hospital, Hangzhou 310022, China; 3 310022 杭州，浙江省肿瘤医院研究所 Research Laboratory, Zhejiang Cancer Hospital, Hangzhou 310022, China

**Keywords:** *BIM*基因, 多态性, 肺肿瘤, 酪氨酸激酶抑制剂, *BIM* Gene, Polymorphism, Lung neoplasms, Tyrosine Kinase Inhibitor

## Abstract

**背景与目的:**

*BIM*基因是BCL-2家族成员之一，是参与细胞死亡的重要介质。在非小细胞肺癌（non-small cell lung cancer, NSCLC）中，BCL-2家族成员蛋白介导的*EGFR*基因突变癌细胞能够激活PI3K/AKT/mTORC和MER/ERT信号通道，决定着细胞的存活或者凋亡。*BIM*基因的BH3域缺失，则容易引起凋亡受阻。本研究通过检测*BIM*基因多态性，探讨其与复治晚期NSCLC表皮生长因子受体酪氨酸激酶抑制剂（epidermal growth factor receptor-tyrosine kinase inhibitor, EGFR-TKI）治疗疗效的关系。

**方法:**

入选2009年1月1日-2012年10月1日就诊于浙江省肿瘤医院的123例复治晚期NSCLC患者，所有患者既往均接受过化疗，失败后接受吉非替尼或厄洛替尼靶向治疗。采用多聚酶链反应方法检测患者外周血白细胞中*BIM*基因多态性。采用SPSS 13.0统计软件分析。

**结果:**

在疾病控制率上，*BIM*基因无多态性的患者较*BIM*基因有多态性的患者呈略好趋势（DCR 75.5% *vs* 57.1%, *χ*^2^=2.931, *P*=0.087）。单因素分析中位PFS，女性长于男性（6.9个月*vs* 4.5个月，*χ*^2^=7.077，*P*=0.008)；不吸烟者长于有吸烟史者（8.0个月*vs* 2.5个月，*χ*^2^=15.277，*P* < 0.001)；病理类型为腺癌的长于其它类型（7.0个月*vs* 2.0个月，*χ*^2^=14.978，*P* < 0.001)；*BIM*基因无多态性的患者中位PFS长于*BIM*基因有多态性的患者（6.0个月*vs* 3.5个月，*χ*^2^=7.035，*P*=0.008)。多因素分析结果显示吸烟、病理类型、*BIM*基因多态性为影响PFS的预后因素。*BIM*基因型与不良反应差异无统计学意义（*P*>0.05）。

**结论:**

*BIM*基因多态性的有无对复治晚期NSCLC EGFR-TKI治疗患者的中位无进展时间有统计学差异，检测患者*BIM*基因多态性对复治晚期NSCLC EGFR-TKI治疗患者的评估预后有重要意义。

## Abstract (en)

**Background and objective:**

*BIM* gene is a member of the BCL-2 family, is involved in cell death. The aim of this study is to explore the relationship between *BIM* gene polymorphism and therapeutic efficacy in the retreatment advanced non-small cell lung cancer (NSCLC) with tyrosine kinase inhibitor (EGFR-TKI).

**Methods:**

In the study, there were 123 patients who were diagnosed with advanced NSCLC in Zhejiang Province Cancer Hospital bewteen January 2009 to October 2012, all of who were received gefitinib and erlotinib therapy after failure to chemotherapy. We detected the genotype of peripheral blood leukocytes of patients with *BIM* gene polymorphism though polymerase chain reaction (PCR). Statistical analysis was performed by SPSS version 13.0.

**Results:**

On the disease control rates, *BIM* gene with no polymorphism type was slightly better trend than polymorphism types in disease control rate DCR (75.5% *vs* 57.1%, *χ*^2^=2.931, *P*=0.087). Univariate analysis the median PFS, women were longer than men (6.9 months *vs* 4.5 months, *χ*^2^=7.077, *P*=0.008). Non-smokers were longer than smokers (8.0 months *vs* 2.5 months, *χ*^2^=15.277, *P* < 0.001). Adenocarcinoma were longer than others pathological type (7.0 months vs 2.0 months, *χ*^2^=14.978, *P* < 0.001). The median PFS in *BIM* gene with no polymorphism type were longer than with polymorphism type (6.0 months *vs* 3.5 months, *χ*^2^=7.035, *P*=0.008). Multi-factor analysis showed that smoking, pathological type, the *BIM* gene polymorphism were the independent prognostic factors for PFS.

**Conclusion:**

The patients with the *BIM* gene no polymorphism have longer the median progression-free time than the polymorphism types in retreatment advanced non-small cell lung cancer patients with tyrosine kinase inhibitor.

目前，肺癌在全球的发病率和死亡率均居恶性肿瘤首位，其中80%为非小细胞肺癌（non-small cell lung cancer, NSCLC)，大部分患者在确诊时已是晚期^[1]^。BR.21^[2]^、TITAN^[3]^和INTEREST^[4]^临床研究显示了厄洛替尼和吉非替尼在晚期NSCLC二三线治疗中的疗效。基于上述大规模的前瞻性多中心临床研究，表皮生长因子受体酪氨酸激酶抑制剂（epidermal growth factor receptor-tyrosine kinase inhibitor, EGFR-TKI）已被批准用于晚期NSCLC的二三线治疗，并且NCCN指南指出在二线及二线以上治疗时，可以不依据肿瘤基因的突变状态。然而，由于个体的差异和肿瘤的异质性，在二三线靶向治疗中，EGFR-TKI的疗效各异。近年来有研究发现血标本中细胞凋亡相关基因多态性与NSCLC及其它恶性肿瘤的治疗疗效及预后有密切的相关性。在恶性肿瘤的发病机制中细胞凋亡机制缺陷占有重要的地位。*BCL*-2基因家族是细胞凋亡的进程中重要的死亡决策者。目前已发现的*BCL*-2基因家族成员超过25种^[4]^：根据其结构和功能可以分为三类：第Ⅰ类具有抗凋亡作用，包括BCL-2、BCL-XL、BCL-W等。第Ⅱ类具有促凋亡作用，包括BAX、BAK等，包含3个BCL-2同源结构域BH1、BH2、BH3。第Ⅲ类具有促凋亡作用并且只含有BH3结构域，包括BID、BAD、BIM、BIK等。这些BCL-2家族基因所编码的蛋白可以形成同源或异源二聚体或多聚体，聚集在线粒体膜上而形成孔道来破坏膜的稳定性，引起细胞色素C（cytochrome C, CytC）的释放并最终导致细胞凋亡的发生。

*BIM*基因作为BCL-2家族成员之一，其编码的蛋白BIM的全称为BCL-2 interaetion mediator of cell death，是参与细胞死亡的重要介质。其最初是由Hsu^[5]^等在用酵母二元杂交的方法筛选与MCL-1相互作用蛋白质时，从卵巢cDNA文库中发现的一种蛋白，都只含有BH3结构域，并且和MCL-1、BCL-2及BCL-XL等抗凋亡因子相互作用而诱导凋亡，因此将其基因命名为BCL-2相关卵巢死亡基因（BCL-2-related ovarian death gene），简称BOD。在人类基因组中，*BIM*基因位于2q12-q13 ^[6]^。在NSCLC中，BCL-2家族成员蛋白介导的*EGFR*基因突变癌细胞能够激活PI3K/AKT/mTORC和MER/ERT信号通道，决定着细胞的存活或者凋亡。*BIM*基因的BH3域缺失，则容易引起凋亡受阻^[7]^。

Ng等^[8]^研究发现在*BIM*基因上存在缺失多态性。在慢性粒细胞白血病（chronic myeloid leukemia, CML）中，*BIM*基因多态性患者对EGFR-TKI（伊马替尼）治疗疗效劣于无*BIM*基因多态性者（*P*=0.02）。在*EGFR*基因突变的NSCLC患者中，*BIM*基因多态性患者对EGFR-TKI的疗效也劣于无*BIM*基因多态性者（*P*=0.027），而使用BH3类似物，则可以逆转对EGFR-TKI的耐药。Nakagawa^[9]^研究报道东方人群在*EGFR*基因突变的NSCLC患者中，*BIM*基因多态性率占12.9%，并且有*BIM*基因多态性的肺癌细胞对吉非替尼的疗效较BIM无多态性的疗效差，当吉非替尼与BH3类似物HDAC抑制剂联合可以逆转因*BIM*基因多态性对EGFR-TKI的耐药。

本文对*BIM*基因多态性与复治晚期NSCLC EGFR-TKI治疗疗效的相关性进行研究。

## 1 材料与方法

### 1.1 研究对象

本研究入选的123例晚期NSCLC患者，于2009年1月1日-2012年10月1日入住浙江省肿瘤医院化疗科，均经细胞病理学或组织病理学确诊为Ⅲb期或Ⅳ期的NSCLC，治疗前经CT扫描证实有可测量病灶，所有患者既往接受过一至二线的化疗，失败后均接受吉非替尼或厄洛替尼的二三线靶向治疗。入组标准：①年龄18岁-80岁；②经组织病理学或细胞病理学证实的Ⅲb期或Ⅳ期NSCLC患者；③有可测量或可评估的病灶；④能随访，依从性好；⑤所有患者既往均接受过一至二线的化疗，失败后均接受吉非替尼或厄洛替尼靶向治疗；⑥签署知情同意书。排除标准：符合以下任意一条标准者，均不得入选：①仅有不可测量病灶者；②严重或未控的内科疾患或感染；③既往或同时合并其它恶性肿瘤；④精神异常。

### 1.2 实验主要试剂、耗材

血液基因组中量提取试剂盒、2×LAmp MasterMix（含染料）、1 kbp Ladder、TAE Buffer（电泳缓冲液）（康为世纪公司）、*BIM*基因上游引物、*BIM*基因下游引物（上海基康生物技术有限公司）、西班牙琼脂糖（西班牙）。

### 1.3 实验主要器材

4 ℃冰箱（MPR-311D，SANYO，日本），-20 ℃冰箱（MDF-330，SANYO，日本），-70 ℃超低温冰箱（MDF-3826W，SANYO，日本），低温高速电动离心机（Neofue 23R，上海力申科学仪器有限公司），漩涡混合器（XW-80A，上海医大仪器厂），PCR仪器、电泳槽、成像仪（Bio-Rad公司，美国），紫外分光光度仪（ND-1000，Nano Drop，美国），各种规格移液器（eppendorf3111，EPPENDORF，德国），各种规格Eppendorf管、8联管、96孔板（Axygen，美国）。

### 1.4 实验步骤

#### 1.4.1 标本采集

收集123例入组的所有患者血标本，在采集血标本之前均告知患者知情同意，并签知情同意书，均在服用靶向药物开始之前留取。抽取外周静脉全血2 mL，置入乙二胺四乙酸钠（EDTA）抗凝管，4 ℃冰箱短时间存放。用离心机低速离心（3, 000 rpm, 10 min），然后分离白细胞层，置于-70 ℃超低温冰箱贮存备用。

#### 1.4.2 外周血DNA提取

采用康为世纪公司的血液基因组中量提取试剂盒（1 mL-5 mL）BloodGen Midi Kit，按照说明书从所留取的肺癌全血标本中提取DNA。

#### 1.4.3 引物设计

按照引物设计的原则，参照文献设计引物序列^[9]^。由上海基康生物有限公司制备。上游引物：5’-AATACCACAGAGGCCCACAG-3’，下游引物：5’-GCCTGAAGGTGCTGAGAAAG-3’。

#### 1.4.4 PCR反应体系

在超净工作台冰盒上进行加样：2×LAmp MasterMix（含染料）12.5 μL，上游引物1 μL（浓度均为10 μM），下游引物1 μL（浓度均为10 μM），DNA 2 μL，去离子水8.5 μL。本实验已设空白对照孔，每个样本均设置复孔。

#### 1.4.5 PCR反应条件

96 ℃预变性30 s，94 ℃变性15 s，60 ℃退火60 s，68 ℃ 10 min延伸，进行29个循环，68 ℃ 20 min终延伸。4 ℃取出，低温保存，备用。

#### 1.4.6 琼脂糖凝胶电泳体系

将TAE缓冲液、1%琼脂糖、溴化乙锭（0.5 μg/mL）在电压120 V的琼脂糖凝胶电泳体系电泳60 min。

### 1.5 疗效评价标准

疗效评定采用实体瘤疗效评价标准（Response Evaluation Criteria in Solid Tumors, RECIST）^[10]^，分为完全缓解（complete response, CR）、部分缓解（partial response, PR）、稳定（stable disease, SD）和进展（progressive disease, PD）。客观缓解率（objective response rate, ORR）=（CR +PR）/（CR+PR+SD+PD）×100%。疾病控制率（disease control rate, DCR）＝（CR+PR+SD）/（CR+PR+SD+PD）×100%。

### 1.6 随访和生存分析

随访采用门诊、电话或书信方式，末次随访时间为2012年11月30日。无进展生存期（progression-free survival, PFS）定义为患者自接受吉非替尼或厄洛替尼靶向治疗开始至疾病进展、死亡或不良反应不可耐受。对于在随访截止日期无进展的病例，在统计时作截尾数据处理。

### 1.7 不良反应评定标准

不良反应按照NCI-CTC3.0，抗癌药物常见不良反应分级标准^[11]^评定，分为0度-4度。

### 1.8 统计学方法

应用SPSS 13.0软件进行统计分析。疗效及不良反应的关系采用*Pearson* *χ*^2^法，单因素分析采用*Kaplan*-*Meier*法及*Log*-*rank*检验，多因素分析采用*Cox*回归。*P* < 0.05为差异有统计学意义。

## 2 结果

### 2.1 患者一般临床特征

患者的一般资料，入组患者共123例，所有患者既往经过一至两线化疗失败后开始二三线EGFR-TKI靶向治疗，其中共48例患者服用吉非替尼（易瑞沙）250 mg，每天1次口服治疗：75例患者服用厄洛替尼（特罗凯）150 mg每天1次口服治疗。所有患者具体临床特征详见（表 1）。

**1:** 患者的一般临床特征 General clinical characteristics of the patients

Variable	*n*	%
Targeted drug		
Gefitinib	48	39.0
Erlotinib	75	61.0
Gender	123	
Female	62	50.4
Male	61	49.6
Age (yr)		
> 60	41	33.3
≤60	82	66.7
Smoking history		
Yes	44	35.8
No	79	64.2
Disease stage		
IV	102	82.9
IIIb	21	17.1
Pathological type		
Adenocarcinoma	97	78.9
Other	26	21.1
Line		
Second line	77	62.6
Third line	46	37.4
Performance status score		
1	96	78.0
≥2	27	22.0

### 2.2 基因多态性检测结果判定

因*BIM*基因多态性，经扩增后目的片段长度无多态性的野生型纯合子型为4, 226 bp，有多态性的包括缺失型和混合型，缺失型纯合子型为1, 323 bp，混合型为4, 226 bp和1, 323 bp两个条带都有（图 1）。

**1:**
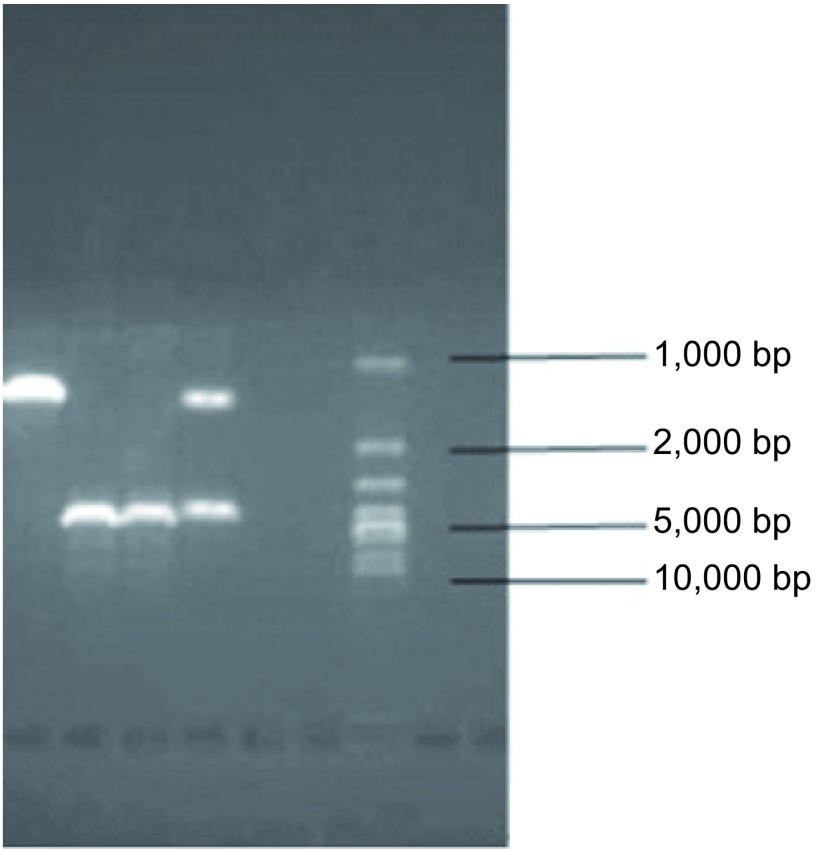
*BIM*基因多态性检测结果。在凝胶成像系统扫描中最右侧为maker电泳通道，（左边数起）第1通道为*BIM*基因缺失型是1, 323 bp条带，2、3通道为野生型是一条4, 226 bp长条带，4通道为混合型是4, 226 bp和1, 323 bp两个条带都有。 *BIM* gene polymorphism detection result diagram. The rightmost of the gel imaging system is maker electrophoresis channel, (left in the cases) the first passage is *BIM* gene deletion whose bands is 1, 323 bp, channel 2 and 3 is the wild-type whose band is 4, 226 bp long, channel 4 is mixed-type having 4, 226 bp and 1, 323 bp two bands.

### 2.3 *BIM*基因多态性与EGFR-TKI治疗疗效的关系

所有样本共123例，其中*BIM*基因无多态性，即野生型的患者有102例，疗效评价无CR患者，PR 33例，SD 44例，PD 25例，ORR为32.4%，DCR为75.5%；BIM有多态性的患者21例，其中缺失型3例，混合型患者18例，无CR患者，PR 3例，SD 9例，PD 9例，ORR为14.3%，DCR为57.1%。在客观缓解率上，*BIM*基因无多态性对比BIM多态性的患者（ORR: 32.4% *vs* 14.3%, *χ*^2^=2.746, *P*=0.098），差异无统计学意义；在疾病控制率上，*BIM*基因无多态性对比BIM多态性的患者（DCR: 75.5% *vs* 57.1%, *χ*^2^=2.931, *P*=0.087），差异无统计学意义（表 2）。

**2:** *BIM*基因多态性与EGFR-TKI治疗疗效的关系 Relationship between *BIM* gene polymorphism and therapeutic efficacy of tyrosine kinase inhibitor

Therapeutic evaluation	*BIM* no polymorphism	*BIM* polymorphism	*χ*^2^	*P*
CR	0	0	0	0
PR	33	3	2.746	0.098
SD	44	9	0.006	0.981
PD	25	9	2.931	0.087
ORR	32.4%	14.3%	2.746	0.098
DCR	75.5%	57.1%	2.931	0.087
CR: complete response; PR: partial response; SD: stable disease; PD: progressive disease; ORR: objective response rate; DCR: disease control rate.				

### 2.4 单因素分析PFS

本研究单因素分析中位PFS，女性长于男性（6.9个月*vs* 4.5个月，*χ*^2^=7.077，*P*=0.008)；不吸烟者长于有吸烟史者（8.0个月*vs* 2.5个月，*χ*^2^=15.277，*P* < 0.001）；病理类型为腺癌长于其它类型（7.0个月*vs* 2.0个月，*χ*^2^=14.978，*P* < 0.001)；*BIM*基因为无多态性中位PFS长于*BIM*基因有多态性的患者（6.0个月*vs* 3.5个月，*χ*^2^=7.035，*P*=0.008)，差异均有统计学意义，而其余临床特征差异均无统计学意义（*P*>0.05）（表 3、图 2）。

**3:** 单因素分析PFS结果 Results of univariate analysis of PFS

Variable	PFS (median)	*χ*^2^	*P*
Targeted drug		0.086	0.769
Gefitinib	5.0		
Erlotinib	4.8		
Gender		7.077	0.008
Female	6.9		
Male	4.5		
Age (yr)		0.119	0.731
> 60	5.1		
≤60	4.9		
Smoking history		15.277	< 0.001
Yes	2.5		
No	8.0		
Disease stage			
Ⅳ	4.7	0.527	0.468
Ⅲb	4.9		
Pathological type		14.978	< 0.001
Adenocarcinoma	7.0		
Other	2.0		
Line		6.684	0.407
Second line	4.8		
Third line	5.3		
Performance status score		0.000	0.983
1	5.0		
≥2	4.9		
*BIM* gene		7.035	0.008
No polymorphism	6.0		
Polymorphism	3.5		
PFS: progression-free survival.			

**2:**
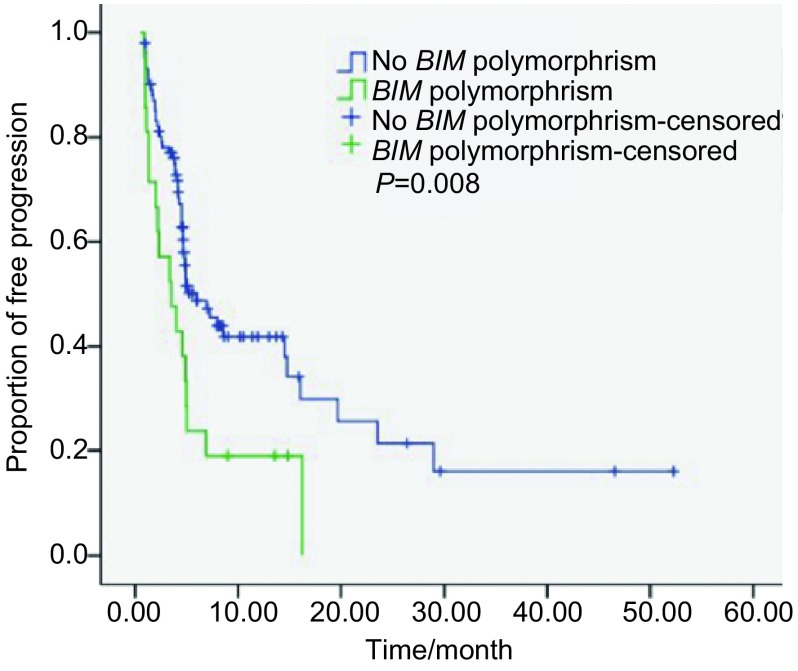
单因素分析PFS与*BIM*基因多态性的关系 Single factor analysis of the relationship between PFS and *BIM* gene polymorphism

### 2.5 多因素分析PFS

多因素分析结果显示吸烟与否（*P*=0.030）、病理类型（腺癌与非腺癌，*P*=0.001）以及*BIM*基因多态性有无（*P*=0.037）为PFS的独立预后因素，其余均为非独立预后因素（表 4）。

**4:** 多因素分析 Multi-factor analysis

	Wald	Sig.	Exp(B)	95%CI for Exp(B)
Targeted drug	0.626	0.429	1.247	0.721-2.157
Disease stage	0.687	0.407	1.328	0.679-2.599
Gender	0.163	0.686	0.846	0.376-1.902
Age	0.048	0.827	1.060	0.630-1.781
Smoking history	4.718	0.030	0.412	0.185-0.917
Pathological type	11.324	0.001	2.739	1.523-4.926
Line	1.940	0.164	0.681	0.396-1.169
Performance status score	0.647	0.421	0.779	0.424-1.431
*BIM* polymorphism	4.362	0.037	0.548	0.311-0.964

### 2.6 毒性反应

本研究结果显示吉非替尼及厄洛替尼治疗NSCLC中，不良反应中主要为皮疹和腹泻。*BIM*基因无多态性Ⅰ度-Ⅳ度皮疹62例，占60.9%，Ⅲ度-Ⅳ度皮疹10例，占9.8%。BIM有多态性者Ⅰ度-Ⅳ度皮疹12例，占57.1%，Ⅲ度-Ⅳ度皮疹2例，占9.5%。*BIM*基因无多态性者Ⅰ度-Ⅳ度腹泻34例，占33.3%，Ⅲ度-Ⅳ度腹泻9例，占8.7%，有多态性者Ⅰ度-Ⅳ度腹泻5例，占23.8%，Ⅲ度-Ⅳ度腹泻1例，占4.7%，差异均无统计学意义（*P*>0.05）。其余少见的不良反应如乏力、间质性肺炎、肝功能损害等差异也均无统计学意义（*P*>0.05）（表 5）。

**5:** *BIM*基因多态性与毒副作用的关系分析 Analysis of the relationship between *BIM* gene polymorphism and side effects

Toxic reaction	Ⅰ-Ⅳ degree (*n*)		*χ*^2^	*P*	Ⅲ-Ⅳ degree (*n*)		*χ*^2^	*P*
	No polymorphism	Polymorphism			No polymorphism	Polymorphism		
Rash	62	12	0.096	0.756	10	2	0.133	0.716
Diarrhea	34	5	0.730	0.393	9	1	0.033	0.856
Feeble	18	3	0.003	0.957	5	1	0.280	0.597
Interstitial pneumonia	3	0	0.000	0.985	1	0	0.208	0.249
Liver function damage	5	1	0.280	0.597	1	0	0.208	0.249

## 3 讨论

*BIM*基因是BCL-2家族的促凋亡基因成员之一，其表达的蛋白属于仅含BH3域蛋白（BH3-only protein），即前凋亡蛋白，广泛表达于正常细胞，在造血细胞的内环境稳定、防止自身免疫及肿瘤的发生中有着极其重要的作用。在NSCLC及其它恶性肿瘤的靶向治疗中，多项研究表明*BIM*基因与靶向治疗的疗效密切相关。

Li等^[12]^研究结果证实，对吉非替尼药物敏感的NSCLC细胞株中，吉非替尼通过BIM蛋白表达数量明显增加来增强对肿瘤细胞的杀伤作用，用RNA干扰技术沉默*BIM*基因后，原来敏感的肿瘤细胞则对吉非替尼耐药，而通过影响BIM信号通道上的PI3K抑制剂和MEK抑制剂联合治疗，能够增加BIM蛋白的表达，从而克服对吉非替尼的耐药。说明*BIM*基因在靶向治疗药物介导的肿瘤细胞凋亡过程中具有十分重要的作用。贺韦东^[13]^研究NSCLC细胞株的凋亡以及促凋亡蛋白BIM的表达，探讨吉非替尼对其的影响。研究的细胞株为H1975的第20外显子T790M突变，第21外显子2573氨基酸位L858R突变，H1650细胞株缺失19外显子缺失。吉非替尼诱导NSCLC细胞株凋亡，其中对H1650、H1975突变株的诱导凋亡率高于A549细胞株（*P* < 0.01），H1650的BIM表达随吉非替尼浓度的增加而增强，H1975的BIM表达较高，而A549的BIM表达需要更高浓度的吉非替尼作用。结果表明吉非替尼可以使NSCLC细胞EGFR突变株细胞内的BIM蛋白表达增高，并且可能通过上调BIM的表达来诱导肿瘤细胞凋亡。Gong^[14]^研究*EGFR*基因突变的NSCLC对EGFR-TKI的耐药机制。其中EGFR-TKI药物对于*EGFR*基因突变的细胞作用引起凋亡通过激活凋亡caspase途径，其中前凋亡蛋白BIM参与其凋亡过程密切相关。厄洛替尼诱导突变的细胞凋亡通过上调BIM水平，而在耐药的细胞株中则BIM低表达。在体外研究通过RNA干扰技术沉默*BIM*基因则引起靶向耐药，而BH3的类似物ABT-737能够增加厄洛替尼介导细胞凋亡的敏感性。

Faber等^[15]^结果表明在*EGFR*突变的NSCLC患者EGFR-TKI治疗中，BIM高表达中位PFS较低表达的长（*P*=0.001）。24例患者中19例接受EGFR-TKI一线治疗，其余接受二线治疗，15例患者BIM高表达，9例BIM低表达，BIM的水平与*EGFR*基因突变类型无相关性。按照RECIST其中PR患者13例，CR患者1例，客观缓解率为64%，BIM低表达患者肿瘤瘤体平均缩小29%，而BIM高表达患者肿瘤瘤体平均缩小57%（*P*=0.04）。中位PFS在低表达患者短于BIM高表达患者。

本研究所有样本共123例，其中*BIM*基因无多态性患者有102例，ORR为32.4%，DCR为75.5%；BIM有多态性的患者21例，缺失型3例，混合型患者18例，ORR为14.3%，DCR为57.1%。在疾病控制率上，*BIM*基因无多态性对比BIM有多态性，差异无统计学意义（DCR: 75.5% *vs* 57.1%, *χ*^2^=2.931, *P*=0.087），*BIM*基因为无多态性较*BIM*基因有多态性患者疾病控制率略好趋势。Katagiri等^[16]^表明*BIM*基因缺失多态性可能是伊马替尼治疗慢性粒细胞白血病的标准。本研究结果首次发现了对于基因突变未明的中国人群的复治晚期NSCLC，*BIM*基因多态性与疗效有相关性趋势。

本研究单因素分析中位PFS，女性长于男性（*P*=0.008)；不吸烟者长于有吸烟史者（*P* < 0.001)；病理类型为腺癌长于其它类型（*P* < 0.001)，差异均有统计学意义。本研究结果与既往如IPASS、OPITIMAL等大型临床研究结果中EGFR-TKI在不吸烟、腺癌患者中疗效较好相类似，是靶向治疗的优势人群。

本研究单因素分析*BIM*基因为无多态性中位PFS长于*BIM*基因有多态性的患者（6.0个月*vs* 3.5个月，*χ*^2^=7.035，*P*=0.008）。本研究结果的多因素分析结果显示*BIM*基因多态性为PFS的独立预后因素。本研究结果进一步从临床中证实了*BIM*基因多态性能够作为预测EGFR-TKI治疗的疗效的指标之一。在*EGFR*基因突变未明的中国患者，*BIM*基因多态性所占的为17.1%，因不同人群不同种族肺癌治疗的不同阶段，可能会存在基因突变的差异，所含基因多态性的比例会有所差别。分析该部分*BIM*基因有多态性的患者缺失了BH3结构域，影响了细胞凋亡通路，对EGFR-TKI疗效欠佳。本研究发现*BIM*基因型与不良反应差异无统计学意义。

本研究为回顾性研究，且样本量有限，有待后续更进一步深入研究及期待前瞻性多中心的大型临床研究为肺癌的治疗提供更高级别的循证医学证据。随着各种基因分析技术不断涌现，将有可能对癌症患者的基因表达谱进行全面高通量分析，用靶基因患者预测抗肿瘤药物的疗效，更好的指导临床个体化用药，使临床用药更具针对性、高效性和安全性。
